# RexAB Promotes the Survival of Staphylococcus aureus Exposed to Multiple Classes of Antibiotics

**DOI:** 10.1128/AAC.00594-21

**Published:** 2021-09-17

**Authors:** Rebecca S. Clarke, Kam Pou Ha, Andrew M. Edwards

**Affiliations:** a MRC Centre for Molecular Bacteriology and Infection, Imperial College London, London, United Kingdom

**Keywords:** *Staphylococcus*, antibiotic, oxidative stress, DNA, DNA repair, AddAB, break, MRSA, repair, SOS system, *Staphylococcus aureus*

## Abstract

Antibiotics inhibit essential bacterial processes, resulting in arrest of growth and, in some cases, cell death. Many antibiotics are also reported to trigger endogenous production of reactive oxygen species (ROS), which damage DNA, leading to induction of the mutagenic SOS response associated with the emergence of drug resistance. However, the type of DNA damage that arises and how this triggers the SOS response are largely unclear. We found that several different classes of antibiotic triggered dose-dependent induction of the SOS response in Staphylococcus aureus, indicative of DNA damage, including some bacteriostatic drugs. The SOS response was heterogenous and varied in magnitude between strains and antibiotics. However, in many cases, full induction of the SOS response was dependent upon the RexAB helicase/nuclease complex, which processes DNA double-strand breaks to produce single-stranded DNA and facilitate RecA nucleoprotein filament formation. The importance of RexAB in repair of DNA was confirmed by measuring bacterial survival during antibiotic exposure, with most drugs having significantly greater bactericidal activity against *rexB* mutants than against wild-type strains. For some, but not all, antibiotics there was no difference in bactericidal activity between wild type and *rexB* mutant under anaerobic conditions, indicative of a role for reactive oxygen species in mediating DNA damage. Taken together, this work confirms previous observations that several classes of antibiotics cause DNA damage in S. aureus and extends them by showing that processing of DNA double-strand breaks by RexAB is a major trigger of the mutagenic SOS response and promotes bacterial survival.

## INTRODUCTION

Staphylococcus aureus is a common cause of both superficial and invasive infections (1). Many of these infections, such as infective endocarditis and osteomyelitis, can be difficult to treat, requiring lengthy courses of therapy (2–10). Staphylococcal infections are also associated with a high rate of relapse and/or the development of chronic infections, even when the bacteria causing the infection appear to be fully antibiotic susceptible (2–10).

There is, therefore, a pressing need to identify new approaches to enhance antibiotic efficacy. To do this, it is important to have a comprehensive understanding of the factors that influence bacterial susceptibility to antibiotics. For example, replication rate has been shown to correlate with susceptibility to several classes of antibiotic (11–13). However, recent evidence suggests that metabolic activity is a better indicator of susceptibility than the replication rate, indicating that metabolism contributes to the bactericidal activity of certain antibacterial drugs (14). This is because the inhibition of bacterial processes by bactericidal antibiotics leads to metabolic perturbations, which in turn result in the generation of reactive oxygen species (ROS) (15–22). These highly reactive molecules damage cellular molecules, including DNA, lipids, and proteins, and have been proposed to contribute to the lethality of bactericidal antibiotics (15, 16, 23–26). However, the magnitude of the damage caused by antibiotic-triggered ROS production and the degree to which these radicals contribute to bacterial killing are unclear (27–29).

DNA damage leads to induction of the SOS response, which involves the expression of genes that encode proteins involved in DNA repair (30–36). In S. aureus, the SOS response includes 16 genes, including RecA and LexA, which are the key regulators of the system (33). It also includes the error-prone polymerase UmuC, the expression of which increases the mutation rate, resulting in increased frequency of antibiotic resistance within populations exposed to SOS-inducing antibiotics and the emergence of the small-colony variant (SCV) phenotype associated with resistance to the oxidative burst of neutrophils and the establishment of chronic infection (33, 37, 38). However, what is not clear is the nature of the DNA damage that is caused by antibiotic-induced ROS or how this triggers the SOS response. This issue is worth resolving because a greater understanding of the mechanisms by which bacteria repair the damage caused by ROS may help to identify new therapeutics that enhance antibiotic activity and reduce the emergence of drug-resistant strains (39). For example, we have shown previously that the combination antibiotic cotrimoxazole (trimethoprim plus sulfamethoxazole) caused DNA double-strand breaks (DSB) and that processing of these by the RexAB nuclease/helicase complex was required for induction of the SOS DNA repair response (30). RexAB is a member of the AddAB family of ATP-dependent helicase/nucleases that process DNA DSBs to produce a 3′ single strand of DNA (34, 39–41). RecA binds to the single-stranded DNA, resulting in a nucleoprotein filament that triggers autocleavage of the LexA transcriptional repressor and induction of the SOS response (39, 41).

The generation of DNA DSBs by cotrimoxazole appeared to be oxygen-dependent, and these were lethal if not repaired, resulting in reduction in CFU counts of a mutant defective for DSB repair (*rexB*::Tn) 50- to 5,000-fold greater than the reduction in CFU counts of wild-type S. aureus (30). However, it was unclear whether DNA DSBs occurred with other antibiotics and if the repair of these by RexAB was a major contributor to induction of the SOS response. If DNA DSBs are a consistent occurrence with diverse antibiotics, then inhibition of RexAB may be an effective way of enhancing the bactericidal activity of antibiotics as well as reducing the emergence of drug-resistant and host-adapted SCV phenotypes.

To test whether our findings with cotrimoxazole were applicable to other antibacterial drugs, we undertook a comprehensive analysis of multiple classes of antibiotics. This revealed that most antibiotics cause DNA damage in S. aureus under aerobic conditions, which appeared to result in DNA DSBs, since mutants lacking DNA DSB repair complex RexAB were more susceptible to antibiotic killing and had reduced induction of the SOS response.

## RESULTS

### Multiple classes of antibiotics cause DNA damage in S. aureus.

DNA damage in most bacteria, including S. aureus, triggers activation of the SOS response, which leads to the transcription of genes whose products contribute to DNA repair (30–34). These genes include *recA*, which encodes the RecA protein required for homologous recombination and, together with LexA, is a key regulator of the SOS response (30–36).

To determine whether antibiotics caused DNA damage in S. aureus, we used a well-characterized P*recA-gfp* reporter system in two distinct genetic backgrounds: SH1000, a methicillin-sensitive S. aureus (MSSA) strain, and JE2, a community-associated methicillin-resistant S. aureus (CA-MRSA) strain of the USA300 lineage (30, 34, 42, 43). This system has been shown to produce a dose-dependent fluorescent response to DNA damage caused by the ROS generator paraquat, mitomycin C, and cotrimoxazole (30, 34).

These SOS reporter strains were then exposed to various classes of clinically relevant antibiotics across a range of concentrations that partially inhibited growth (Fig. S1). These included both bactericidal (cotrimoxazole, ciprofloxacin, nitrofurantoin, oxacillin, daptomycin, gentamicin) and bacteriostatic (chloramphenicol, linezolid) drugs. For most antibiotics, the concentrations ranged from 0.125× to 1× the MIC of the antibiotic. However, for cotrimoxazole, higher concentrations were needed to show growth inhibition (Fig. S1), most likely due to the inoculum effect since a higher concentration of bacteria was used in P*recA* reporter assays than in MIC assays (44). We also used a higher range of concentrations of oxacillin for the USA300 strain because it is resistant to most β-lactams (42).

**FIG 1:**
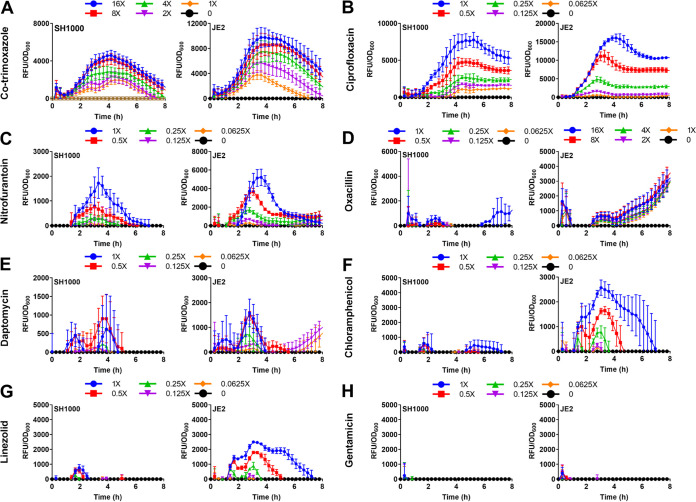
Induction of the SOS response in S. aureus SH1000 and JE2 by diverse classes of antibiotics. (A to H) Induction of SOS measured by GFP expression driven from a P*recA*-*gfp* reporter construct upon exposure to a range of concentrations of various antibiotics. Concentrations were chosen based on their ability to cause growth inhibition and represent multiples of the MIC of the individual strain as indicated in the key above each graph. GFP fluorescence was normalized to OD_600_ to determine induction of SOS relative to cell density. Data represent the mean from 3 independent experiments (*n* = 3). Representative OD_600_ measurements alone are shown in Fig. S1. Error bars represent standard deviation of the mean.

As expected, we found that cotrimoxazole, ciprofloxacin, nitrofurantoin, and oxacillin triggered SOS induction in both the SH1000 and JE2 strains, albeit to various degrees and with different temporal dynamics (Fig. 1A to D) (30, 34–36, 45). In all cases, however, there was evidence of dose-dependent induction of the SOS response (Table S1). DNA damage was also apparent during exposure to the bactericidal lipopeptide antibiotic daptomycin and the bacteriostatic drugs chloramphenicol and linezolid, again with differences in the size and time-dependence of the response between antibiotics and with some differences between the two strains (Fig. 1E to G) (Table S1). However, there was almost no induction of the SOS response during bacterial exposure to gentamicin at any of the concentrations used (Fig. 1H). Taken together, these data indicated that most clinically relevant classes of antibiotics, including bacteriostatic agents, caused DNA damage in S. aureus.

### SOS induction is partly due to processing of DNA double-strand breaks by the RexAB helicase/nuclease complex.

We have shown previously that induction of the SOS response by cotrimoxazole is largely due to the processing of DNA double-strand breaks (DSBs) by the AddAB family RexAB nuclease/helicase complex and the resulting formation of a RecA nucleoprotein filament that leads to the autocatalytic cleavage of LexA (30, 31, 34, 37, 40, 41). Therefore, we determined whether SOS induction by other classes of antibiotics was also due to RexAB-mediated processing of DNA DSBs. As before, cotrimoxazole was included in these assays as a control.

**FIG 2:**
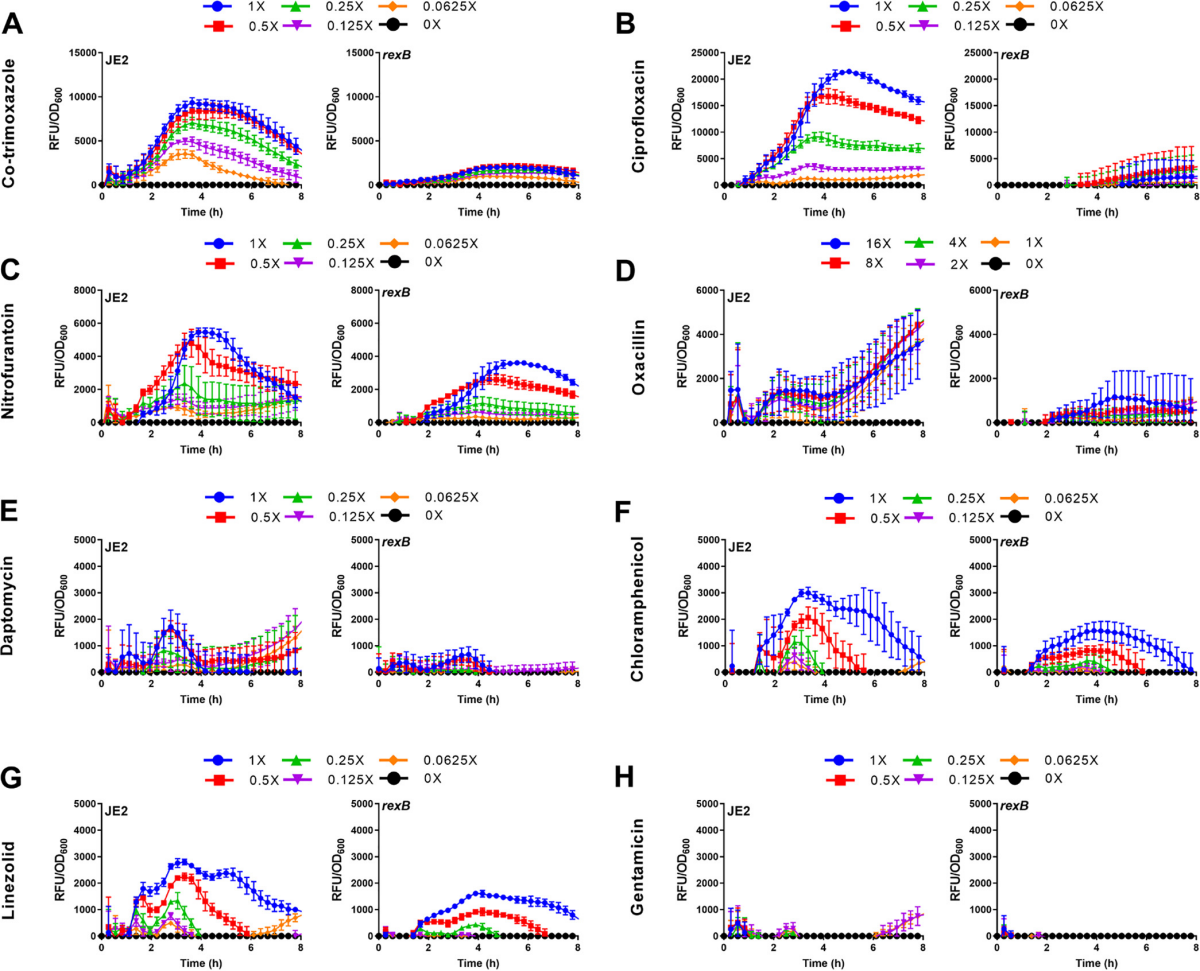
RexAB is required for maximal induction of the SOS response during exposure to antibiotics. (A to H) Induction of SOS response of JE2 wild type and *rexB* mutant measured by GFP expression upon exposure to a range of sublethal concentrations of antibiotics. Concentrations of antibiotic are labeled by multiples of the MIC of the wild-type strain. GFP fluorescence was normalized by OD_600_ to determine induction of SOS relative to cell density (*n* = 3). Representative OD_600_ measurements alone are shown in Fig. S1. Error bars represent standard deviation of the mean.

To do this, we compared green fluorescent protein (GFP) fluorescence from wild-type S. aureus JE2 and a *rexB*::Tn mutant defective for RexAB, both of which contained the P*recA*-*gfp* reporter system, during exposure to the same panel of antibiotics as that described for Figure 1 (30, 34, 38, 39). As expected from our previous work, we found that the lack of RexAB reduced *recA* induction relative to that of the wild type during exposure to cotrimoxazole (Fig. 2A and Table S2) (30). We also observed reduced *recA* expression in the *rexB*::Tn mutant relative to that in the wild type during exposure to the quinolone antibiotic ciprofloxacin, which is known to cause DNA DSBs (Fig. 2B and Table S2) (45).

For nitrofurantoin, oxacillin, daptomycin, chloramphenicol, and linezolid, we also observed lower levels of SOS induction in the *rexB*::Tn mutant relative to those in the wild type, although, while statistically significant, the difference between wild-type and mutant strains was not as large as that for cotrimoxazole and ciprofloxacin (Fig. 2C to H and Table S2). As expected from previous data (Fig. 1G), very little *recA* induction was observed from either wild type or *rexB*::Tn mutant during exposure to gentamicin (Fig. 2G). Therefore, as for cotrimoxazole, RexAB is required for maximal induction of SOS in response to DNA damage caused by several clinically relevant antibiotics, indicating that these drugs cause DNA DSBs in S. aureus.

### DNA DSB repair reduces bacterial susceptibility to several classes of antibiotics.

The requirement of RexAB for maximal induction of the SOS response indicated that exposure to most antibiotics caused DNA DSBs (30, 34). Since DSBs are lethal if not repaired, we hypothesized that mutants defective for RexAB would be more susceptible than wild-type strains to those antibiotics that triggered the SOS response (34, 39, 40).

To test this, we determined the MIC of each antibiotic for wild-type S. aureus SH1000 and JE2 and associated *rexB*::Tn mutants (Table 1). The *rexB* mutants in both JE2 and SH1000 strains were ≥2-fold more susceptible to 7 of the 8 antibiotic-tested conditions (Table 1). Importantly, the absence of RexAB increased the susceptibility of the MRSA strain JE2 to both oxacillin and ciprofloxacin 4-fold, despite this strain being resistant to both antibiotics (42).

**TABLE 1:** MIC values (μg ml^−1^) of S. aureus WT and *rexB* mutant in SH1000 and JE2 backgrounds for various antibiotics (*n* ≥ 3; median MIC is shown); the fold reduction in MIC of the *rexB*::Tn mutants relative to the wild type is also shown

Antibiotic	MIC value of strain:			
	SH1000 WT	SH1000 *rexB*::Tn	JE2 WT	JE2 *rexB*::Tn
Cotrimoxazole	0.25	0.125 (2-fold)	0.5	0.25 (2-fold)
Ciprofloxacin	0.125	0.0078 (16-fold)	16	4 (4-fold)
Nitrofurantoin	8	4 (2-fold)	16	4 (4-fold)
Oxacillin	0.125	0.06 (2-fold)	4	1 (4-fold)
Daptomycin	0.25	0.125 (2-fold)	0.25	0.125 (2-fold)
Chloramphenicol	4	1 (4-fold)	4	2 (2-fold)
Linezolid	1	0.25 (4-fold)	1	0.25 (4-fold)
Gentamicin	0.0625	0.031 (2-fold)	0.125	0.125 (no diff)

The one exception was gentamicin, where the SH1000 *rexB*::Tn mutant was 2-fold more susceptible to the antibiotic, but the JE2 *rexB*::Tn mutant had the same MIC as the wild-type strain, in keeping with the fact that this antibiotic did not trigger the SOS response under the conditions tested (Table 1). Taken together, the MIC data provide additional evidence that most antibiotics cause DNA DSBs in S. aureus.

### RexAB promotes staphylococcal tolerance of several classes of antibiotics.

We have shown previously that DNA DSB repair by RexAB enables staphylococcal tolerance of the combination antibiotic cotrimoxazole (30). Since most of the other antibiotics we examined also appeared to cause DNA DSBs, leading to increased susceptibility of *rexB*::Tn mutants in MIC measurements, we hypothesized that RexAB would also contribute to bacterial survival during exposure to a supra-MIC of these other antibacterial drugs.

**FIG 3:**
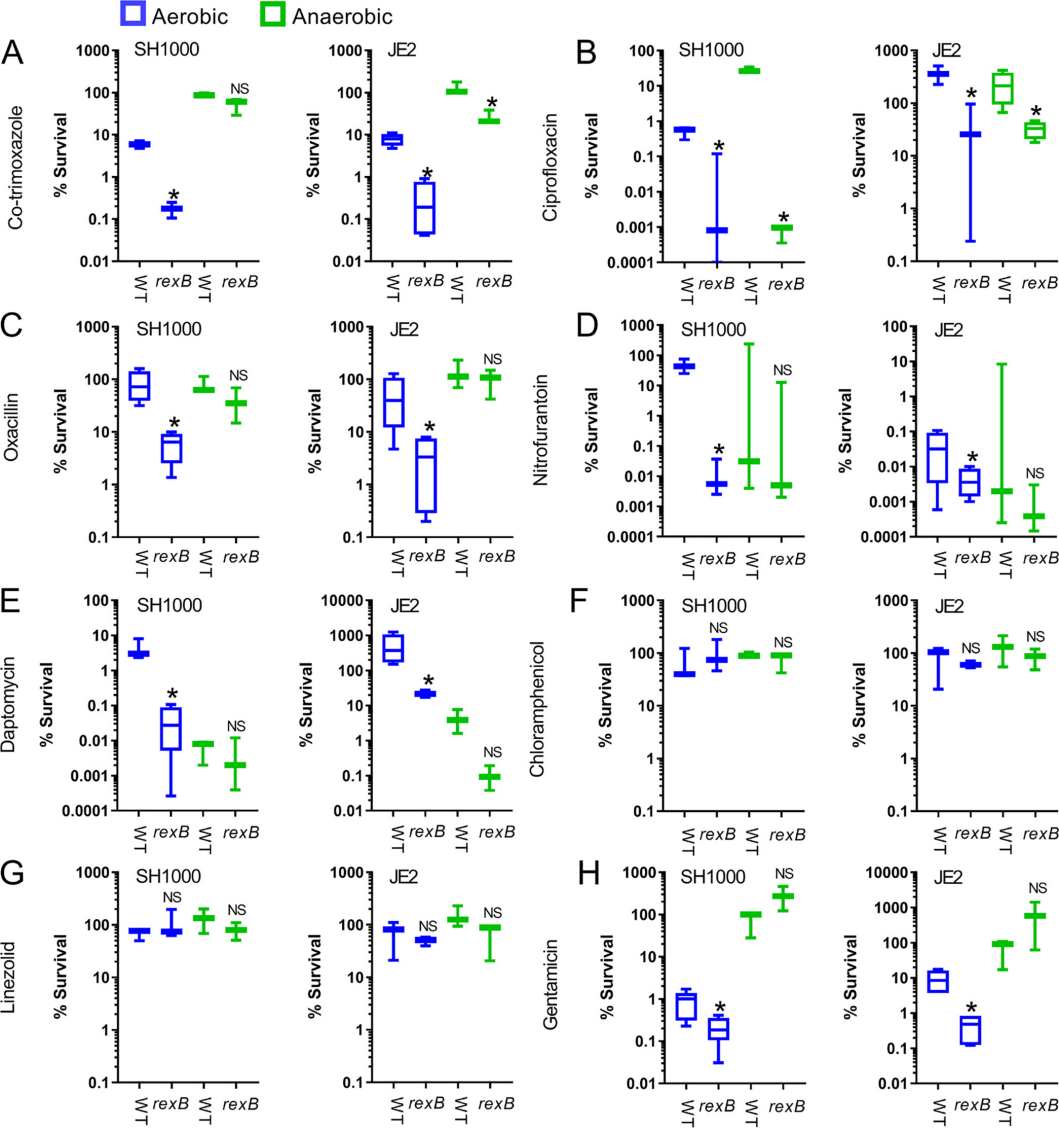
Lack of effective DNA repair increases the killing of S. aureus by bactericidal antibiotics under aerobic conditions. (A to H) Survival of S. aureus wild type (WT) and *rexB*::Tn mutant in SH1000 and JE2 backgrounds after 8 h of incubation at 37°C in TSB supplemented with 10× MIC. Survival was assessed under aerobic (blue) or anaerobic (green) conditions (*n* = 3). Data were analyzed by ordinary one-way ANOVA with Tukey’s correction for multiple comparisons (*, *P* < 0.05 mutant versus wild type under the same atmospheric condition; NS, not significant) and presented as a box and whisker plot with error bars showing the full data range.

To test this, we exposed wild-type S. aureus SH1000 and JE2 and associated *rexB*::Tn mutants to 10× the MIC of the wild type of each of the antibiotics used in previous assays and measured survival after 8 h of incubation at 37°C in an aerobic atmosphere (Fig. 3). Similar to the MIC assays, 6 of 8 antibiotics tested were more active against the *rexB*::Tn mutant than against wild-type bacteria, resulting in lower survival of the DNA repair-defective strains (Fig. 3). The two antibiotics where there was no difference in survival between wild type and *rexB*::Tn mutants were linezolid and chloramphenicol, which are both bacteriostatic and did not reduce CFU counts of any of the strains (Fig. 3F and G). The remaining 6 antibiotics (cotrimoxazole, ciprofloxacin, oxacillin, nitrofurantoin, daptomycin, gentamicin), all of which are classified as bactericidal, caused significantly greater decreases in CFU counts of the *rexB*::Tn mutants than in CFU counts of wild-type bacteria (Fig. 3A, B, C, D, E, and H). The increased susceptibility of the *rexB*::Tn mutants resulted in reductions in CFU counts 5- to 500-fold greater than those of wild-type cells after 8-h exposure to the 6 bactericidal antibiotics (Fig. 3A, B, C, D, E, and H).

The observation that the *rexB*::Tn mutants of both SH1000 and JE2 were killed more efficiently than wild-type strains by gentamicin (Fig. 3H) was surprising given the lack of SOS response during exposure to the aminoglycoside antibiotic (Fig. 1H and 2H). To determine whether these findings were applicable to other aminoglycoside antibiotics, we measured the susceptibility of wild type and *rexB*::Tn mutants to kanamycin. Wild-type JE2 and SH1000, and both of the corresponding *rexB*::Tn mutants, had identical kanamycin MICs (1 μg ml^−1^). Furthermore, there were no differences in survival between wild type and *rexB*::Tn mutant after 8-h exposure to kanamycin at 10× the MIC (Fig. S2), although there were differences in susceptibility between the two strains. Therefore, while gentamicin was more bactericidal against *rexB*::Tn mutants than against wild-type strains, this does not appear to be the case for all aminoglycoside antibiotics.

To test whether DNA DSBs caused by bactericidal antibiotics were due to endogenous ROS production, we repeated bactericidal activity assays under anaerobic conditions. As reported previously, cotrimoxazole lost most of its bactericidal activity against wild-type bacteria in the absence of oxygen, as did ciprofloxacin and gentamicin, the latter due to the reduction in membrane potential in the absence of oxygen (30, 46, 47) (Fig. 3A, B, and H). The increased susceptibility of the *rexB*::Tn mutant to cotrimoxazole and oxacillin relative to that of wild type seen under aerobic conditions was also much less pronounced or absent under anaerobic conditions, suggesting that ROS may contribute to DNA DSBs in the presence of these antibiotics (Fig. 3A and C).

The bactericidal antibiotics nitrofurantoin and daptomycin retained bactericidal activity under anaerobic conditions (Fig. 3C to E). However, nitrofurantoin and daptomycin also retained bactericidal activity against the *rexB*::Tn mutants, suggesting that they caused DNA DSBs in an ROS-independent manner (Fig. 3D and E).

To explore the potential role of endogenous ROS in DNA DSB production further, we measured the production of ROS using a fluorescent dye (30) in S. aureus strains exposed to two antibiotics that were significantly more bactericidal against the *rexB*::Tn mutant under aerobic than under anaerobic conditions (cotrimoxazole and oxacillin) and compared them with two antibiotics that killed the *rexB*::Tn mutant efficiently under both aerobic and anaerobic conditions (nitrofurantoin and daptomycin). Antibiotic concentrations were the same as those used in P*recA-gfp* reporter assays to enable comparison. As expected from previous work (30), exposure to cotrimoxazole caused dose-dependent ROS production in both S. aureus strains (Fig. S3A and Table S3). A similar ROS production profile was seen during oxacillin exposure, but nitrofurantoin and daptomycin did not cause dose-dependent ROS production (Fig. S3B to D and Table S3).

In summary, ROS appear to contribute to DNA DSBs caused by some antibiotics, as evidenced by increased susceptibility of the *rexB*::Tn mutant to several antibiotics relative to that of the wild type under aerobic conditions and dose-dependent ROS production. However, some antibiotics appear to cause DNA DSBs under aerobic conditions that may not be due to ROS, suggesting that additional mechanisms of DNA damage are possible. Furthermore, ROS were not required for the lethality of all antibiotics since some retained bactericidal activity under anaerobic conditions.

### RexAB promotes staphylococcal survival during exposure to oxacillin and fosfomycin.

**FIG 4:**
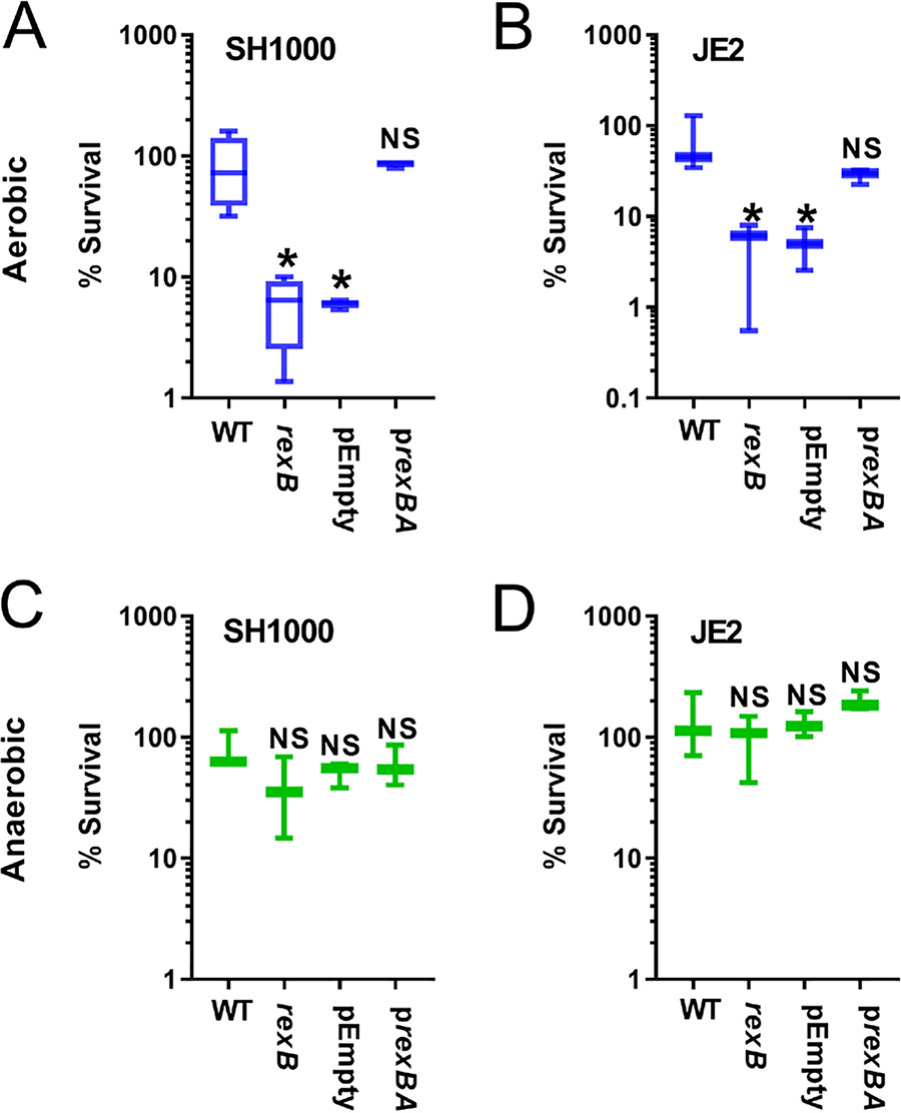
Complementation of the *rexB*::Tn mutant restores tolerance to oxacillin in S. aureus SH1000 and JE2. Survival of S. aureus WT and *rexB* mutant in SH1000 and JE2 backgrounds after 8 h of incubation at 37°C in TSB supplemented with 10× MIC. Survival was assessed under aerobic (blue) or anaerobic (green) conditions (*n* = 3). Data were analyzed by one-way ANOVA with Dunnett’s correction for multiple comparisons (*, *P* < 0.05 versus wild type) and presented as a box and whisker plot with error bars showing the full data range.

The finding that loss of RexAB resulted in increased killing of both the SH1000 MSSA and JE2 MRSA strains by the frontline antistaphylococcal β-lactam oxacillin was particularly noteworthy because this indicated a mechanism by which MRSA strains could be resensitized to the antibiotic. Therefore, we repeated this assay and included mutants complemented with the *rexBA* operon (34) (Fig. 4). As expected, the *rexB*::Tn mutants were more susceptible to killing by oxacillin than were wild-type bacteria under aerobic but not anaerobic conditions. Complementation of mutations with plasmids containing the *rexBA* operon, but not the plasmid alone, restored survival to wild-type levels, confirming the role of RexAB in staphylococcal tolerance of oxacillin (Fig. 4).

To understand whether RexAB promoted staphylococcal survival during exposure to other β-lactams, we exposed wild type and *rexB*::Tn mutants to a panel of other β-lactams at 10× the MIC (Fig. S3 and Table S4). The *rexB*::Tn mutant of S. aureus JE2 was more susceptible to bactericidal effects of penicillin G and imipenem than was the wild type, but it was not more susceptible to the six other β-lactams tested (Fig. S3). In contrast, the *rexB*::Tn mutant of SH1000 was more susceptible than wild type to 6/8 β-lactams (Fig. S3). This difference may reflect the fact that JE2 is a MRSA strain while SH1000 is not. To further explore the susceptibility of the *rexB*::Tn mutant to cell wall-targeting antibiotics, we extended our analysis to vancomycin and fosfomycin. For both JE2 and SH1000 strains, the *rexB*::Tn mutants were more susceptible than wild type to killing by fosfomycin but not by vancomycin. Therefore, the absence of RexAB sensitizes S. aureus to oxacillin and fosfomycin, but these findings do not extend to all antibiotics that target the cell wall, with differences between strains and within antibiotic class.

## DISCUSSION

Staphylococcal infections are associated with high rates of treatment failure, even in the case of apparently drug-susceptible strains (1–10). This, together with the threat posed by multidrug-resistant MRSA strains, necessitates a greater understanding of how antibiotics function and the identification of opportunities to improve their efficacy (9).

There is compelling evidence that diverse antibiotics trigger metabolic perturbations in bacteria that lead to endogenous ROS production under aerobic conditions (14–22). However, the consequences of this for bacterial viability remain a matter of debate (22, 27–29). This is important because if endogenous ROS production is a common property of antibiotics, then it could be exploited to enhance treatment outcomes, for example by designing inhibitors of bacterial processes that detoxify ROS or repair the damage it causes (30, 34, 39).

To understand whether antibiotics cause ROS-mediated damage in S. aureus, we focused on the degree to which antibiotic exposure resulted in bacterial DNA damage, since nucleic acids are frequently attacked by endogenous ROS, and the consequences of that damage for bacterial survival (23–26, 30, 34).

Using a P*recA*-*gfp* reporter assay, we observed that the SOS response in S. aureus was triggered by several different classes of antibiotics, indicative of DNA damage. While this was expected for DNA-targeting antibiotics such as the fluoroquinolone ciprofloxacin (45), SOS induction also occurred with antibiotics that do not directly target bacterial DNA, such as oxacillin, daptomycin, and linezolid. It has been shown that certain β-lactam antibiotics induce the SOS response in Escherichia coli via the DpiBA two-component system rather than via DNA damage (48). Although this mechanism has been hypothesized for S. aureus, our data show that S. aureus *rexB*::Tn mutants in both the JE2 and SH1000 genetic backgrounds were more susceptible to killing by oxacillin, demonstrating that DNA damage does occur during exposure to this β-lactam antibiotic and that this is at least partially responsible for triggering the SOS response. Therefore, our findings are in keeping with work showing that β-lactam antibiotics trigger endogenous ROS production via elevated TCA cycle activity in response to cell wall damage, leading to increased mutation rate (49).

It is important to note that while JE2 is a MRSA strain, it is not highly resistant to β-lactam antibiotics as seen in the case of, e.g., strain COL (50). Therefore, it is not clear whether oxacillin would induce the SOS response in highly resistant MRSA strains. Furthermore, the *rexB*::*Tn* mutant of the JE2 strain was only more susceptible than wild type to a few β-lactam antibiotics, whereas the SH1000 *rexB*::*Tn* mutant was more susceptible than wild type to most of them. This may suggest that resistance to antibiotics provides S. aureus a measure of protection from antibiotic-induced ROS production and DNA damage. The differing sensitivity of the *rexB*::*Tn* mutant to each of the β-lactam antibiotics may also reflect differences in the antibiotics’ affinity for penicillin binding protein 1, which is required for SOS induction (51).

While it is still a controversial topic, there is increasing evidence that many classes of antibiotics trigger the endogenous production of ROS. However, the degree to which these ROS contribute to bactericidal activity is less clear (22, 27–29). Our data provide evidence that many antibiotics cause DNA damage, in part via ROS but also apparently via ROS-independent mechanisms. However, this DNA damage appears to be largely tolerated by wild-type bacteria via RexAB-mediated processing of DSBs, which triggers the SOS response to facilitate repair via homologous recombination. Furthermore, while some antibiotics had greater bactericidal activity under anaerobic conditions, this was not the case for daptomycin or nitrofurantoin.

The production of ROS by bactericidal but not bacteriostatic antibiotics has been proposed to explain the antibiotics’ differences in lethality. However, we observed SOS induction during exposure of S. aureus to the bacteriostatic antibiotics linezolid and chloramphenicol but not to the bactericidal antibiotic gentamicin.

The fact that linezolid and chloramphenicol appeared to trigger the SOS response but not DNA DSBs may be explained by differences in the type of DNA damage caused by each of the antibiotics. While several different types of DNA damage trigger SOS, only those leading to DSBs would be expected to promote susceptibility of the *rexB* mutant (41, 52, 53). As such, it is possible that bactericidal antibiotics trigger the potentially lethal DNA DSBs, while bacteriostatic antibiotics trigger nonlethal types of DNA damage. In keeping with this hypothesis, the absence of RexAB had only a small effect on SOS induction in S. aureus caused by linezolid or chloramphenicol.

It is unclear why gentamicin did not trigger SOS during antibiotic exposure since it appeared to cause DNA DSBs in the antibiotic tolerance assays, because we observed 5- to 10-fold increased susceptibility of RexAB-deficient strains to the aminoglycoside antibiotic in bactericidal killing assays. However, it may be the case that high concentrations of the antibiotic are needed for DNA damage.

Combined, our data indicate differences between antibiotics in the degree of DNA damage caused, as well as the time required to cause damage, and these differences may explain some of the debate around the contribution of ROS to antibiotic-mediated killing. However, the data strongly suggest that DNA DSBs are a common consequence of the exposure of S. aureus to several different classes of antibiotics and that an inability to repair those DSBs increases bacterial susceptibility to several antibacterial drugs. These findings are similar to those reported for E. coli, where mutants defective for DNA DSB repair (defective for *recB* or *recC*) were more susceptible than the wild type to at least 8 different antibiotics (54). Crucially, we found that disruption of DNA DSB repair restored quinolone susceptibility in an otherwise resistant strain of S. aureus, which is also similar to what has been seen in E. coli and Klebsiella pneumoniae (55). We also found that an inability to repair DSBs restored oxacillin susceptibility in the JE2 MRSA strain, although it remains to be seen if this finding is applicable to other MRSA strains, particularly those with high-level resistance to β-lactams.

The identification of RexAB as important for staphylococcal survival during exposure to several different antibiotics, and the fact that loss of RexAB resensitizes otherwise resistant strains to some antibiotics, makes this complex a potential target for novel therapeutics. Crucially, there is a lack of RexAB homologues in eukaryotes, reducing the likelihood of host toxicity (40, 41, 52, 53, 55, 56). Inhibitors of RexAB would be expected to enhance the bactericidal activity of several different classes of antibiotic, as well as reduce the induction of the mutagenic SOS response, which is associated with the emergence of antibiotic resistance and mutants that can resist host immune defenses (37, 38, 56, 57). We have also shown recently that DNA DSB repair is important for staphylococcal resistance to host immune defenses, in keeping with similar findings with several other bacterial pathogens, providing an additional potential benefit of targeting this complex (34, 58–61).

In summary, our data demonstrate that staphylococcal DNA is damaged by several classes of bactericidal antibiotics, which appears to result in DNA DSBs that are processed by RexAB and trigger the SOS response for repair. Therefore, RexAB promotes staphylococcal survival during exposure to multiple antibacterial drugs and is therefore a potential target for novel therapeutics that sensitize S. aureus to antibiotics.

## MATERIALS AND METHODS

### Bacterial strains and culture conditions.

The bacterial strains used in this study are listed in Table 2. S. aureus was cultured in tryptic soy broth (TSB) or Mueller-Hinton broth (MHB) to stationary phase (18 h) at 37°C, with shaking (180 rpm). Media were supplemented with antibiotics as required. For strains with the P*recA*-*gfp* reporter plasmid, kanamycin (90 μg ml^−1^ was included), and for *rexB*::Tn mutants, erythromycin, was added to the medium (10 μg ml^−1^) (30, 34). The p*itet* plasmid integrates stably into the staphylococcal chromosome and did not require selection. To induce expression of the *rexBA* operon in complemented strains, the medium was supplemented with anhydrotetracycline (AHT) at 100 ng ml^−1^.

**TABLE 2:** Bacterial strains used in this study^*a*^

Staphylococcus aureus strain	Description	Source (reference number)
SH1000	*rsbU^+^* derivative of the laboratory strain 8325-4	43
SH1000 *rexB*::Tn	SH1000 with a *bursa aurealis* transposon insertion in *rexB,* Ery^r^	30
SH1000 *rexB*::Tn p*itet* empty	SH1000 with a *bursa aurealis* transposon insertion in *rexB* with integrated p*itet* empty plasmid, Ery^r^ Cam^r^	30
SH1000 *rexB*::Tn p*itet rexAB*	SH1000 with a *bursa aurealis* transposon insertion in *rexB* with integrated p*itet* with AHT-inducible *rexB*, Ery^r^ Cam^r^	30
JE2	A derivative of CA-MRSA USA300 LAC, cured of plasmids	42
JE2 *rexB*::Tn	JE2 with a *bursa aurealis* transposon insertion in *rexB,* Ery^r^	42
JE2 *rexB*::Tn p*itet* empty	JE2 with a *bursa aurealis* transposon insertion in *rexB* with integrated p*itet* empty plasmid, Ery^r^ Cam^r^	30
JE2 *rexB*::Tn p*itet rexAB*	JE2 with a *bursa aurealis* transposon insertion in *rexB* with integrated p*itet* with AHT-inducible *rexB*, Ery^r^ Cam^r^	30
JE2 pCN34 *PrecA-gfp*	JE2 containing pCN34 with *gfp* under the control of the *recA* promoter, Kan^r^	30
JE2 *rexB*::Tn pCN34 *PrecA-gfp*	JE2 *rexB*::Tn containing pCN34 with *gfp* under the control of the *recA* promoter, Ery^r^ Kan^r^	30

a Ery^r^, Cam^r^, and Kan^r^ indicate the presence of resistance markers for erythromycin, chloramphenicol, and kanamycin, respectively.

### *recA*-*gfp* fluorescent reporter assay.

As described previously (30, 34), promoter-reporter gene constructs in JE2 and SH1000 backgrounds were used to quantify expression of *recA*. Antibiotic 2-fold dilutions were made in flat-bottomed, black-walled, 96-well plates containing TSB and inoculated with 1/10 dilution of a stationary-phase culture of the reporter strains. Plates were placed into an Infinite M200-PRO microplate reader (Tecan) where cultures were grown for 17 h at 37°C (700 rpm), and both absorbance at 600 nm (OD_600_) and GFP relative fluorescence units (RFU) were measured every 30 min. To account for differences in cell density, RFU values were normalized by OD_600_ data at each time point.

### Determination of MIC.

MICs were determined using a serial broth dilution protocol as described previously (30, 62). Bacteria were diluted to 1 × 10^5^ CFU ml^−1^ and incubated in flat-bottomed 96-well plates with a range of antibiotic concentrations for 17 h at 37°C under static conditions (aerobic, anaerobic, or 5% CO_2_). Medium containing daptomycin was supplemented with 1.25 mM CaCl_2_. The MIC was defined as the lowest concentration at which no growth was observed.

### Antibiotic survival assay.

Bacteria were adjusted to 10^8^ CFU ml^−1^ in TSB (S. aureus) supplemented with antibiotics at 10× MIC. For aerobic incubation, 3 ml of medium was inoculated in 30-ml universal tubes and incubated with shaking at 180 rpm. For anaerobic conditions, 6 ml of prereduced medium in 7-ml bijou tubes was inoculated and incubated statically in an anaerobic cabinet. Cultures were incubated at 37°C, and bacterial viability was determined by CFU counts. Culture medium containing daptomycin was supplemented with 1.25 mM CaCl_2_. Survival was calculated as a percentage of the number of bacteria in the starting inoculum.

### Endogenous ROS production.

ROS production was detected and quantified using 2′,7′-dichlorodihydrofluorescein diacetate (H_2_DCFDA) using a 96-well-based assay as described previously (30). Bacteria (∼3.33 × 10^8^ CFU ml^−1^) were incubated in TSB with shaking at 37°C. The growth medium was supplemented with 25 μM H_2_DCFDA and antibiotics at various concentrations, and OD_600_ and fluorescence (excitation, 495 nm; emission, 525 nm) were quantified every 1,000 s (∼17 min). Fluorescence data were normalized against OD_600_ values to account for differences in bacterial growth between antibiotic concentrations.

### Statistical analyses.

Data are represented as the mean or median from three or more independent experiments and analyzed by Student’s *t* test or one-way or two-way analysis of variance (ANOVA) corrected for multiple comparisons, as described in the figure legends. For each experiment, “*n*” refers to the number of independent biological replicates. *P* < 0.05 was considered significant between data points. Statistical analyses and area under the curve values were calculated using GraphPad Prism 7 for Windows.
