# Supplementary material for: RexAB Promotes the Survival of Staphylococcus aureus Exposed to Multiple Classes of Antibiotics
Source: Antimicrob Agents Chemother. 2021 Sep 17;65(10):e00594-21. doi: 10.1128/AAC.00594-21 (PMC8448105; doi:10.1128/AAC.00594-21)
Supplement: Supplemental file 1 — Supplemental material. Download AAC.00594-21-s0001.pdf, PDF file, 0.3 MB [file aac.00594-21-s0001.pdf]

Supplementary information file

Multiple classes of antibiotics cause DNA double strand breaks in *Staphylococcus aureus*

Rebecca S. Clarke[1], Kam Pou Ha[1,2] and Andrew M. Edwards[1#]

[1] MRC Centre for Molecular Bacteriology and Infection, Imperial College London, Armstrong Rd, London, SW7 2AZ, UK.

[2] Present address: Université Paris-Saclay, CEA, CNRS, Institute for Integrative Biology of the Cell (I2BC), 91198, Gif-sur-Yvette, France.

Supplementary figures S1, S2, S3 and supplementary table S1.

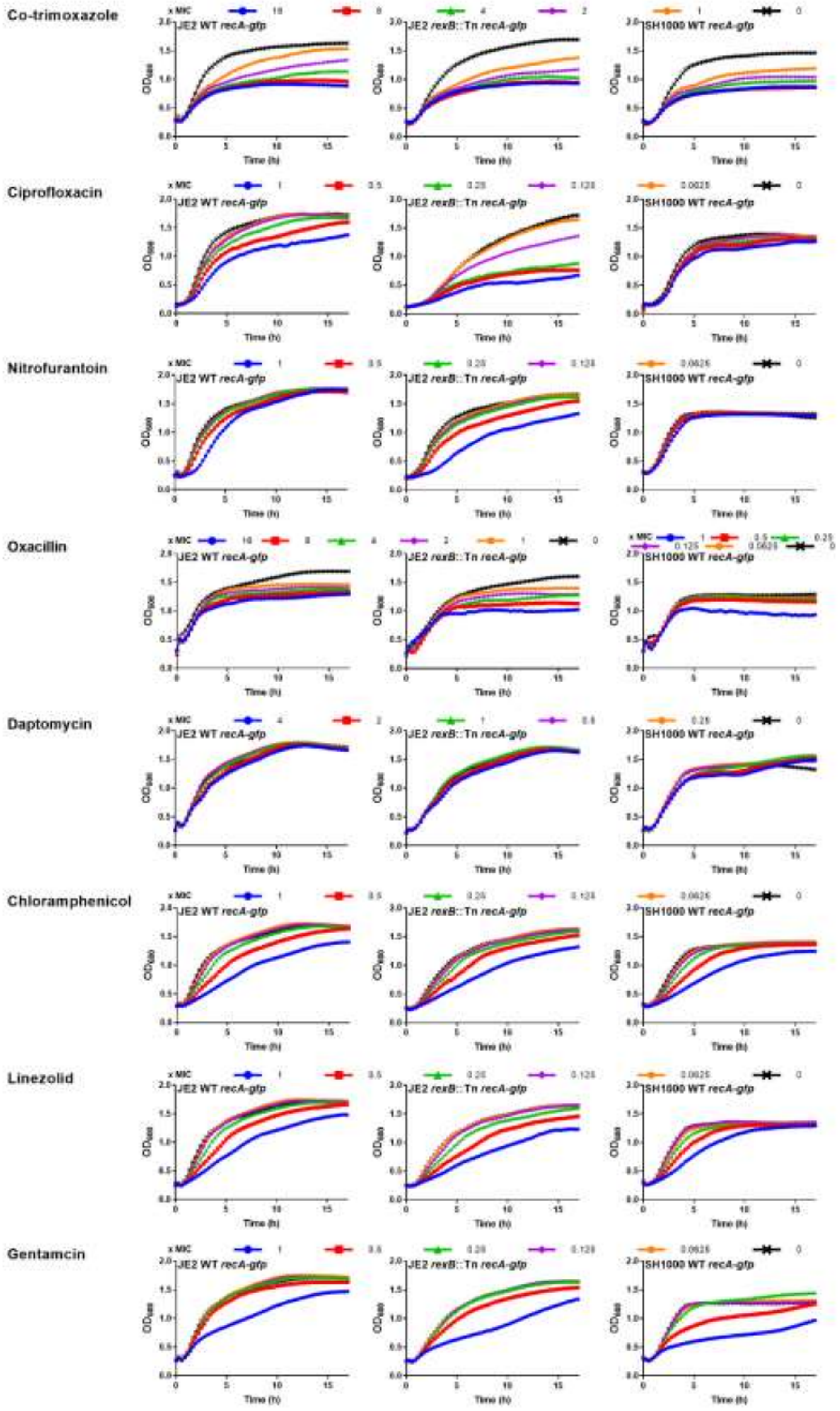

Supplementary figure S1. Representative graphs showing magnitude of growth inhibition of *S. aureus* at concentrations of antibiotics used for Fig. 1 and Fig. 2. Concentrations of antibiotics are labelled by multiple of the MIC of the individual WT strain (x MIC). Error bars were omitted for clarity.

Antibiotic	Wild type JE2					
	0X	0.0625X	0.125X	0.25X	0.5X	1X
	0	**48463±2545**	**58806±1643**	**69755±2220**	**84003±2419**	**86562±4391**
Co-trimoxazole	Wild type SH1000					
	0X	0.0625X	0.125X	0.25X	0.5X	1X
	0	**14356±611**	**24819±929**	**35647±1075**	**39609±1206**	**43322±945**
	Wild type JE2					
	0X	0.0625X	0.125X	0.25X	0.5X	1X
	0	**7643±373**	**11603±420**	**33767±649**	**85349±909**	**135760±752**
Ciprofloxacin	Wild type SH1000					
	0X	0.0625X	0.125X	0.25X	0.5X	1X
	0	**12888±512**	**20591±594**	**34171±848**	**56438±1007**	**82586±1798**
	Wild type JE2					
Nitrofurantoin	0X	0.0625X	0.125X	0.25X	0.5X	1X
	0	**6521±708**	**6911±780**	**11217±787**	**15060±781**	**24458±1482**
	Wild type SH1000					
	0X	0.0625X	0.125X	0.25X	0.5X	1X
	0	110±58	359±107	**594±106**	**1826±139**	**4085±270**
	Wild type JE2					
Oxacillin	0X	1X	2X	4X	8X	16X
	0	**45228±385**	**47506±555**	**44467±487**	**47217±851**	**42380±801**
	Wild type SH1000					
	0X	0.0625X	0.125X	0.25X	0.5X	1X
	0	**4650±610**	**6015±795**	**5574±777**	**5785±601**	**25246±861**
	Wild type JE2					
Daptomycin	0X	0.0625X	0.125X	0.25X	0.5X	1X
	0	**5899±464**	**7203±407**	**8346±1000**	**13013±505**	**25701±893**
	Wild type SH1000					
	0X	0.0625X	0.125X	0.25X	0.5X	1X
	0	3145±818	**1504±674**	**9861±1075**	**7030±938**	**24200±1152**
	Wild type JE2					
Chloramphenicol	0X	0.0625X	0.125X	0.25X	0.5X	1X
	0	**12765±395**	**25048±487**	**49213±1101**	**67622±1523**	**60626±1583**
	Wild type SH1000					
	0X	0.0625X	0.125X	0.25X	0.5X	1X
	0	63±47	36±44	0	0	**399±270**
	Wild type JE2					
Linezolid	0X	0.0625X	0.125X	0.25X	0.5X	1X
	0	0	152±24	**961±87**	**3986±148**	**9409±209**
	Wild type SH1000					
	0X	0.0625X	0.125X	0.25X	0.5X	1X
	0	0	0	**85±28**	**370±65**	**564±81**
	Wild type JE2					
Gentamicin	0X	0.0625X	0.125X	0.25X	0.5X	1X
	0	**87±39**	**140±75**	0	0	0
	Wild type SH1000					
	0X	0.0625X	0.125X	0.25X	0.5X	1X
	0	**40±68**	0	0	0	0

Supplementary table S1. Area under the curve values for the data presented in figure 1 for each strain and each concentration (multiple of MIC) of each antibiotic. Values significantly different ($p = <0.05$) from no antibiotic controls are indicated in bold. Data were analysed by one-way ANOVA with Dunnett's post hoc test.

Antibiotic	Wild type JE2					
Co-trimoxazole	0X	0.0625X	0.125X	0.25X	0.5X	1X
	0	36441±2525	53178±1015	64776±1809	81406±2659	87196±1506
	rexB::Tn					
	0X	0.0625X	0.125X	0.25X	0.5X	1X
	0	**21467±490**	**20296±841**	**25690±1412**	**30278±1466**	**33037±1404**
	Wild type JE2					
Ciprofloxacin	0X	0.0625X	0.125X	0.25X	0.5X	1X
	0	21752±618	40835±740	85103±1077	139617±1491	186440±1762
	rexB::Tn					
	0X	0.0625X	0.125X	0.25X	0.5X	1X
	0	**5796±540**	**11573±928**	**34577±4033**	**37517±6144**	**19083±3957**
	Wild type JE2					
Nitrofurantoin	0X	0.0625X	0.125X	0.25X	0.5X	1X
	0	14836±326	16876±961	16070±1350	28939±1096	32036±901
	rexB::Tn					
	0X	0.0625X	0.125X	0.25X	0.5X	1X
	0	**3096±234**	**6450±248**	**7547±484**	**21144±587**	32850±482
	Wild type JE2					
Oxacillin	0X	1X	2X	4X	8X	16X
	0	45539±1098	56732±1335	60616±1304	63976±1737	58082±1971
	rexB::Tn					
	0X	1X	2X	4X	8X	16X
	0	**8717±1322**	**10333±759**	**8281±1002**	**5945±939**	**10155±1793**
	Wild type JE2					
Daptomycin	0X	0.0625X	0.125X	0.25X	0.5X	1X
	0	17737±892	21013±1235	13159±2076	14492±1207	17716±1610
	rexB::Tn					
	0X	0.0625X	0.125X	0.25X	0.5X	1X
	0	**2028±308**	**2991±380**	**8352±1300**	**8808±506**	**14778±752**
	Wild type JE2					
Chloramphenicol	0X	0.0625X	0.125X	0.25X	0.5X	1X
	0	8727±650	9928±759	30780±1124	45747±1207	47598±1126
	rexB::Tn					
	0X	0.0625X	0.125X	0.25X	0.5X	1X
	0	**4718±259**	9001±317	**18431±437**	**30381±484**	**35556±562**
	Wild type JE2					
Linezolid	0X	0.0625X	0.125X	0.25X	0.5X	1X
	0	7237±386	3125±307	2233±171	5876±179	13847±212
	rexB::Tn					
	0X	0.0625X	0.125X	0.25X	0.5X	1X
	0	**31±12**	**47±23**	**727±55**	**3278±101**	**8040±133**
	Wild type JE2					
Gentamicin	0X	0.0625X	0.125X	0.25X	0.5X	1X
	0	6465±291	5749±505	193±98	0	0
	rexB::Tn					
	0X	0.0625X	0.125X	0.25X	0.5X	1X
	0	**22±16**	**53±51**	0	0	0

Supplementary table S2. Area under the curve values for the data presented in figure 2 for each strain and each concentration (multiple of MIC) of each antibiotic. Values significantly different (p = <0.05) between the wild type and *rexB* mutant are indicated in bold. Data were analysed by two-way ANOVA with Sidak's post hoc test.

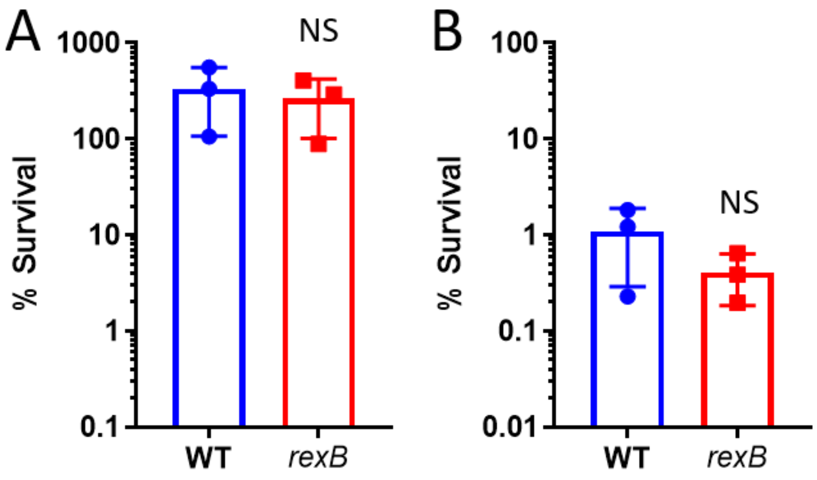

Supplementary figure S2. Survival of *S. aureus* JE2 (A) and SH1000 (B) after exposure to kanamycin. Survival of each strain was determined by CFU counts after 8 h exposure to kanamycin at 10X the MIC. There was no significant difference in survival between wild type and *rexB* mutant for either strain (p = >0.05 by Student's TTest).

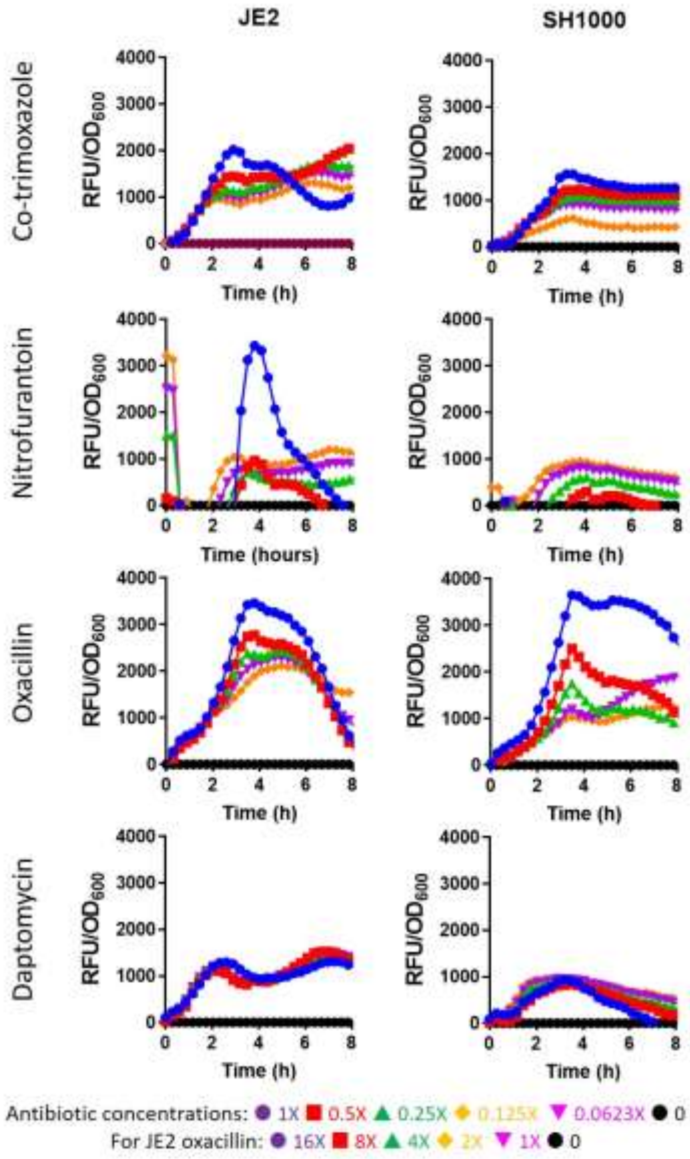

Supplementary Figure S3. Antibiotic-triggered ROS production in *S. aureus*. ROS production was determined by reactivity with the DCF fluorophore and adjusted for differences in bacterial growth (OD_{600}) due to the presence of the antibiotics. For all assays bar JE2 with oxacillin, bacteria were exposed to a range of antibiotic concentrations that related to the MIC (0.125-1X for all assays except JE2 and oxacillin, where bacteria were exposed to a range of concentrations up to and including 16X the MIC). Data represent the mean of 3 independent experiments. Error bars were omitted for clarity.

Antibiotic	Wild type JE2					
Co-trimoxazole	0X	0.0625X	0.125X	0.25X	0.5X	1X
	0	**4746±246**	**5234±267**	**5670±265**	**6242±301**	**7216±411**
	Wild type SH1000					
	0X	0.0625X	0.125X	0.25X	0.5X	1X
	0	**2191±394**	**3827±238**	**4411±272**	**4781±285**	**5658±391**
	Wild type JE2					
Nitrofurantoin	0X	0.0625X	0.125X	0.25X	0.5X	1X
	0	**23036±846**	**18612±838**	**14493±1151**	**10716±2026**	**20441±3652**
	Wild type SH1000					
	0X	0.0625X	0.125X	0.25X	0.5X	1X
	0	**8809±476**	**5407±412**	**6187±466**	**13431±988**	**24785±2003**
	Wild type JE2					
Oxacillin	0X	1X	2X	4X	8X	16X
	0	**8490±370**	**9135±423**	**9813±585**	**10571±771**	**13113±1096**
	Wild type SH1000					
	0X	0.0625X	0.125X	0.25X	0.5X	1X
	0	**14693±737**	**23448±1665**	**49888±3603**	**60038±1672**	**68697±6480**
	Wild type JE2					
Daptomycin	0X	0.0625X	0.125X	0.25X	0.5X	1X
	0	**23959±333**	**25273±337**	**24958±369**	**25099±401**	**16595±548**
	Wild type SH1000					
	0X	0.0625X	0.125X	0.25X	0.5X	1X
	0	**7682±349**	**6604±312**	**6520±241**	**7703±521**	**12854±833**

Supplementary table S3. Area under the curve values for the data presented in supplementary figure S3 for each strain and each concentration (multiple of MIC) of each antibiotic. Values significantly different (p = <0.05) between bacteria not exposed to antibiotic (0X) and each of the concentrations of antibiotic are indicated in bold. Data were analysed by one-way ANOVA with Dunnett's post hoc test.

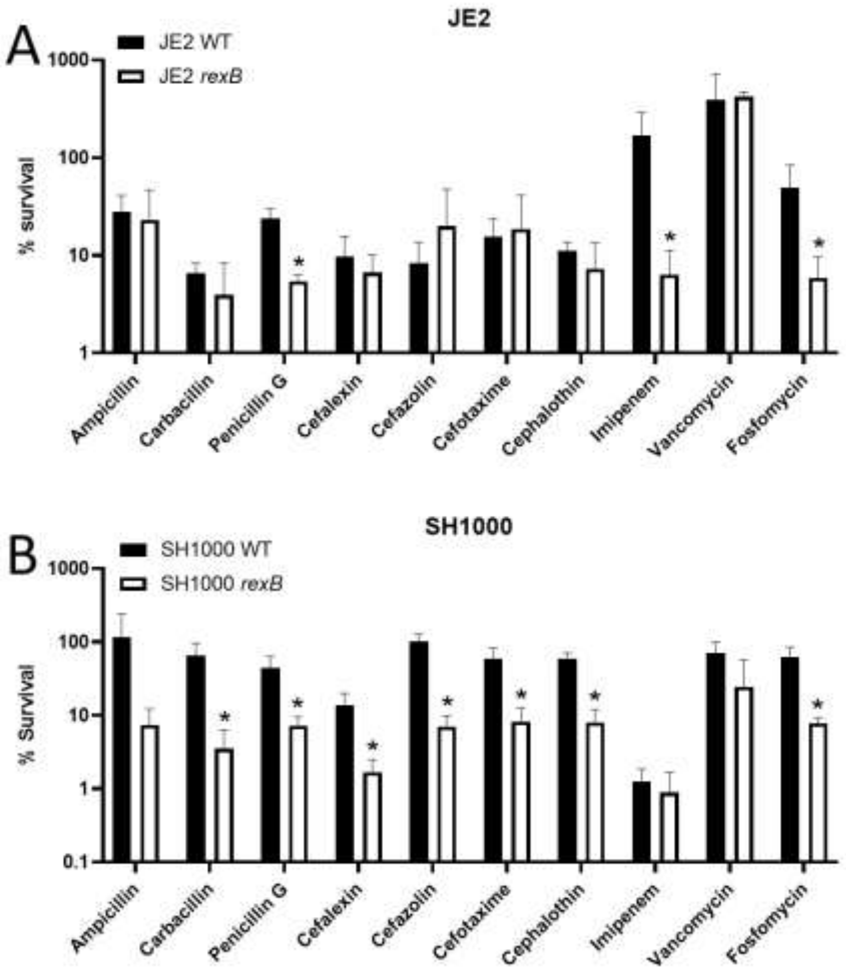

Supplementary figure S4. Survival of *S. aureus* JE2 (A) and SH1000 (B) after exposure to various antibiotics that target the bacterial cell wall. Survival of wild type (WT) or *rexB* mutant of each strain was determined by CFU counts after 8 h exposure to the indicated antibiotics at 10X the MIC (see Table S1 below). Significant differences in survival between wild type and *rexB* mutant for either strain are indicated (*, p = >0.05 by Student's TTest wild type versus *rexB* mutant).

Class	Penicillins			Cephalosporins				Carbapenem	Glycopeptide	Fosfomycin
Antibiotic	Ampicillin	Carbacillin	Penicillin G	Cefalexin	Cefazolin	Cefotaxime	Cephalothin	Imipenem	Vanomycin	Fosfomycin
JE2	40	40	12.5	40	10	40	5	5	10	320
SH1000	10	20	0.625	20	2.5	10	5	5	5	40

Supplementary Table S4. Concentrations (µg ml^{-1}) of antibiotics used in bactericidal activity assay experiments presented in supplementary figure S4. This are 10 X MIC of each antibiotic for wild type *S. aureus* JE2 or SH1000.
